# Serum Albumin as a Prognostic Marker in Geriatric Intermediate Care: A Retrospective Cohort Study

**DOI:** 10.7759/cureus.97422

**Published:** 2025-11-21

**Authors:** Zane Attard, Simon Attard, Matthew Vella, Andrea Calleja, Luke Meli, Mark Spiteri, Antoine Vella

**Affiliations:** 1 Internal Medicine, Mater Dei Hospital, Msida, MLT; 2 Geriatrics, Saint Vincent de Paul Residence, Luqa, MLT

**Keywords:** albumin, geriatric medicine, intermediate care, prognosis, prognostic marker

## Abstract

Purpose: This study aimed to evaluate the prognostic value of admission serum albumin levels on six-month mortality and readmission in older adults admitted to an intermediate care facility. We hypothesized that lower albumin would be independently associated with adverse outcomes.

Methods: We conducted a retrospective cohort study at Saint Vincent de Paul Residence, including 145 intermediate care inpatients and 134 community-dwelling outpatient controls between April 2024 and March 2025. Data included demographics, frailty scores, comorbidities, CRP and albumin. Logistic regression assessed the relationship between albumin and outcomes, adjusting for age, sex, CRP, frailty and multimorbidity.

Results: Mean admission albumin was 29.8 g/L in inpatients versus 40.0 g/L in controls (p<0.0001). Six-month readmission and mortality rates were 34.5% and 36.6%, respectively. Albumin independently predicted adverse composite outcomes (OR 0.439 per 5 g/L increase, p 0.003).

Conclusion: Low serum albumin is an independent predictor of six-month mortality and adverse outcomes in frail older adults admitted to intermediate care.

## Introduction

The advancing age of the general population, in the advent of modern medical knowledge, poses a significant challenge to healthcare systems worldwide. These challenges put older adults at risk, highlighting the need for developing targeted and tailored approaches for this vulnerable cohort of patients. Several prognostic factors have been found to place older adults at an increased risk of mortality, such as weight fluctuations [1] and sarcopenia [2].

The first intermediate care ward in Malta saw its inception in March 2024. This ward was designed to act as an alternative acute admission pathway for frail older adults, bypassing the acute general hospital. This ward’s main goal is to provide hospital-level care in a holistic environment, led by geriatricians, to community-dwelling older adults admitted from their own homes or care homes. 

Serum albumin is a widely available biomarker reflecting nutritional status, inflammation, and overall disease burden. Hypoalbuminemia is common in hospitalized patients and has been associated with poor outcomes, including longer stays and increased mortality. In older adults, low albumin correlates with frailty and worse survival [3]. However, its prognostic role in alternative acute geriatric admission pathways such as intermediate care wards remains unclear. We aimed to evaluate whether admission albumin predicts six‑month mortality and morbidity among acutely admitted intermediate care patients, comparing these outcomes to an age‑matched community‑dwelling cohort. We hypothesized that hypoalbuminemia would be strongly associated with a poorer prognosis.

## Materials and methods

Study design

We conducted a retrospective cohort study at the Intermediate Care Ward of Saint Vincent De Paul Residence, Malta, from April 2024 to March 2025. This ward is Malta’s first facility providing acute medical care to older adults outside the general hospital, with a multidisciplinary geriatric focus. A control cohort of outpatients who underwent serum albumin measurement during the same period was included for comparison. Data collection and extraction were performed between March 2025 and June 2025. The study utilized routinely collected clinical and laboratory data, with no direct patient contact.

Study setting

The intermediate care ward is an acute medical admission unit within a geriatric hospital setting, designed to provide comprehensive multidisciplinary care for older adults aged 65 years and older presenting with acute medical illness not requiring surgical intervention. The ward operates on principles of geriatric medicine within a calm, holistic environment that prioritizes functional and psychosocial outcomes alongside acute medical management. Daily multidisciplinary input is provided by geriatricians, occupational therapists, physiotherapists, and nursing staff who collaborate in structured ward rounds and clinical meetings. This model represents an alternative acute admission pathway, allowing appropriate triaging of frail older adults away from traditional intensive acute hospital settings while maintaining access to acute medical care. The ward's geriatric-focused, person-centered approach emphasizes early mobilization, comprehensive assessment, functional recovery and discharge planning, with the goal of optimizing health outcomes and quality of life in this vulnerable population.

Study population and eligibility

Inclusion Criteria (Inpatients)

Inclusion criteria for inpatients were adults aged ≥65 years admitted to the Intermediate Care Ward with serum albumin measured within 24 hours of admission and with six‑month follow‑up data for readmission and mortality.

Inclusion Criteria (Outpatients)

Inclusion criteria for outpatients were adults aged ≥65 years with an outpatient serum albumin measurement during the study period and no inpatient admission at the time of sampling.

Exclusion Criteria

Exclusion criteria were missing data, duplicated records, patients still admitted at the time of data collection, insufficient follow-up period and patients with a terminal diagnosis.

Study size and sampling

All consecutive eligible inpatients were included (n = 145). A convenience sample of outpatients (n = 134) was selected from the laboratory database during the same period. As a retrospective exploratory analysis, no a priori power calculation was performed; this is acknowledged as a limitation.

Variables and data sources

Demographics (age, sex), C‑reactive protein (CRP, mg/L), serum albumin (g/L), Clinical Frailty Scale (CFS, 1-9), admission source, primary diagnosis, length of stay (days), multimorbidity (defined as ≥3 chronic conditions), six‑month all‑cause readmission, and six‑month all‑cause mortality were extracted from electronic health records and laboratory systems. Outcomes were coded as binary variables (yes/no).

Outcomes

The primary outcome was serum albumin concentration, compared between inpatients and outpatients. Secondary outcomes included all-cause hospital readmission within six months of discharge and all-cause mortality within six months of discharge.

Statistical analysis

Continuous variables were summarized as mean or median (interquartile range) as appropriate, categorical variables as n (%). Between‑group comparisons used an independent‑samples t‑test or Mann-Whitney U test for continuous variables and a chi‑square test for categorical variables. Serum albumin differences were also assessed using multivariable linear regression adjusted for age, sex, CRP, and CFS. For inpatient outcomes, logistic regression estimated the association between albumin (per 5 g/L increase) and the composite endpoint. Models were adjusted for age, sex, CRP, CFS, multimorbidity, and length of stay where appropriate, with reporting guided by available events. Two‑sided P values < 0.05 were considered statistically significant. Analyses were conducted in Python 3.11 using pandas (v2.2.2) and statsmodels (v0.14.0). No imputation was performed for missing data; records with missing key fields were excluded. For inpatient outcomes, six-month readmission and six-month mortality were modelled using logistic regression. Due to event counts and stability considerations, univariable models with albumin (per 5 g/L increase) were reported. All statistical analyses were two-sided, with significance defined as p<0.05. Analyses were performed in Python 3.11 using the pandas (v2.2.2) and statsmodels (v0.14.0) libraries.

Ethics

Ethics approval was obtained from the local ethics board. All data were anonymized prior to analysis, and no patient contact occurred. This study was conducted in accordance with the principles of the Declaration of Helsinki.

## Results

A total of 145 inpatients from the intermediate care ward met the inclusion criteria. The mean age was 85.7 years on admission. A total of 134 outpatients were included as a control group, with a mean age of 80.5 years. Mean serum albumin was 29.8 g/L in the inpatient cohort and 40.0 g/L in the outpatient cohort. Further cohort demographics are available in Tables 1, 2. 

**Table 1:** Demographic data of the inpatient cohort. CFS: Clinical Frailty Scale

Variable	Value
Age mean (years)	85.7
Gender (% female)	63.4
CFS median (range 1-9)	7
CRP mean (mg/L) (range 0-6 mg/L)	77.6
Albumin mean (g/L) (range 33-45 g/L)	29.8
Length of stay (LOS) mean (days)	20
Most common diagnosis	Respiratory disease (49 patients)

**Table 2:** Demographic data of the control group.

Variable	Value
Age mean (years)	80.5
Gender (% female)	50.0
CRP mean (mg/L) (range 0-6 mg/L)	2.2
Albumin mean (g/L) (range 33-45 g/L)	40.0

From the inpatient cohort, 50 (34.38%) patients were readmitted either to the intermediate care ward or main hospital within the follow up six-month period and 53 (36.6%) patients passed away during the follow up six-month period. Mean serum albumin on admission was significantly lower in the inpatient cohort compared to the outpatient control group (29.8 g/L vs 40.0 g/L). After adjustment for age, sex, and CRP in a multivariable regression model, this difference remained highly statistically significant (p <0.0001). 

A multivariable logistic regression analysis was performed to assess the degree of effect of albumin on composite six-month outcomes independently of other variables measured in this study (Table 3). Albumin was found to be a strong predictor of adverse outcomes in our cohort of patients, even after adjusting for other variables (p 0.003, odds ratio (OR) 0.439). For every 5 g/L rise in albumin, the odds of an adverse outcome were found to be reduced by 56.1%. 

**Table 3:** Composite six-month event (readmission or mortality) adjusted logistic regression. Multivariable logistic regression analysis was performed. A p-value less than 0.05 was considered statistically significant. CFS: Clinical Frailty Scale; LOS: length of stay

Variable	Odds Ratio	p-Value	CI 97.5%
Albumin (per 5 g/L) (range 33-45 g/L)	0.439	0.003	0.754
Age (per year)	1.023	0.421	1.082
Male sex	1.409	0.428	3.291
CRP (mg/L) (range 0-6 mg/L)	0.872	0.401	1.201
CFS (per point) (range 0-9)	1.117	0.615	1.716
LOS (per day)	0.997	0.780	1.021
Multimorbidity	0.951	0.899	2.056

Outcomes were also assessed by albumin category (Table 4), defined as normal (≥35 g/L), mild (31-34 g/L), moderate (25-30 g/L) and severe (<25g/L) hypoalbuminemia. Chi-squared tests demonstrated a statistically significant association between albumin category and both six-month mortality (p <0.0001) and composite outcome of readmission or death (p 0.0089) but not with six-month readmission alone (p 0.082). 

**Table 4:** Six‑month outcomes by admission albumin category. The chi-square test was performed. A p-value less than 0.05 was considered statistically significant.

Albumin Group	n	Readmission (%)	Mortality (%)	Composite (%)
Normal (≥35 g/L)	41	31.7	19.5	51.2
Mild (31-34 g/L)	42	50.0	19.0	69.0
Moderate (25-30 g/L)	28	25.0	57.1	78.6
Severe (<25 g/L)	34	26.5	61.8	85.3
Overall p-value (χ²)	/	0.082	<0.0001	0.0089

## Discussion

In this retrospective cohort of geriatric intermediate care patients, we found that admission serum albumin levels were a strong independent prognostic marker for six-month outcomes. Patients with hypoalbuminemia had significantly higher mortality and composite event rates (death or readmission) compared with those with normal albumin levels. Importantly, this relationship remained robust after adjusting for age, sex, CRP, frailty, and multimorbidity, highlighting that albumin provides prognostic information beyond these established risk factors. Furthermore, a clear dose-response gradient was observed: risk of adverse outcomes rose progressively from mild to moderate to severe hypoalbuminemia, consistent with the stepwise increase in mortality described in previous studies [3,4]. Compared to outpatient controls, intermediate care inpatients experienced substantially worse six-month outcomes, reflecting the heightened vulnerability of this subgroup.

Our findings align with and extend previous evidence linking low serum albumin to poor outcomes in older adults across diverse care settings. Tang et al. examined 512 elderly hip-fracture patients and reported that admission albumin < 35 g/L was associated with approximately a two-fold increase in 30-day readmission (adjusted OR 2.12, p = 0.003) compared with normal levels. They also demonstrated a dose-response pattern (p < 0.001) in readmission risk with decreasing albumin levels [5]. Similarly, Huang et al., in a cohort of 2,387 hip-fracture patients followed for an average of 37 months, showed that each 1 g/L increase in albumin was associated with a 5% reduction in all-cause mortality risk (HR 0.95, 95% CI 0.93-0.97) [6]. In another large Italian study of 2,780 hospitalized adults aged ≥ 65 years, hypoalbuminemia independently predicted higher in-hospital mortality (adjusted OR 2.31, 95% CI 1.76-3.04), longer length of stay, and worse discharge outcomes [7]. Likewise, a Japanese longitudinal study of 600 community-dwelling adults aged ≥ 70 years followed for 10 years found that individuals with hypoalbuminemia had a 2.7-fold higher mortality risk after adjustment for confounders, and that overall mortality decreased by 16% per 1 g/L increase in albumin [8]. Hypoalbuminemia was found to be more prevalent in adults aged ≥ 65 years in an Italian prospective population-based study. Interestingly, lower albumin levels were associated with a greater risk of mortality in adults aged ≥ 65 years but not in younger individuals [9]. This further reinforces the use of albumin levels as a prognostic tool in older individuals. 

Abraham et al. conducted a prospective study in a surgical center involving 150 patients aged 65 and over, finding that hypoalbuminemia predicted longer inpatient stays and increased complications, though it did not reach statistical significance for higher mortality or readmission rates. Notably, their sample was younger, with an average age of 77 and a shorter average LOS of 4 days, compared to our older cohort with an average age of 87 years and an average LOS of 20 days. These differences may reflect a more robust and less comorbid group, yet serum albumin maintained its predictive value for poorer inpatient outcomes [10]. Recent literature encompassing both hospitalized and community-dwelling older adults confirmed that hypoalbuminemia is consistently associated with increased mortality across both settings and that those with greater functional limitations in activities of daily living tend to have lower serum albumin levels [11-13]. Hypoalbuminemia is also associated with reduced functional mobility in older adults possibly reflective of the reduced physiological reserve associated with aging [13]. Collectively, these studies reinforce our findings that serum albumin is a robust and independent prognostic biomarker among older adults. However, our study adds novel evidence specific to intermediate care settings, which are underrepresented in the literature. Most previous investigations have focused on surgical or acute hospital populations with shorter follow-up periods (typically ≤ 30 days). In contrast, our study examined a frail, medically complex cohort with a longer follow-up (6 months), showing that the prognostic value of albumin persists in this context. Moreover, by adjusting for frailty (CFS) and multimorbidity, our findings extend beyond earlier research that did not control for these geriatric-specific factors. The observed dose-response trend further supports the biological plausibility of albumin as a graded risk indicator.

The mechanisms underlying hypoalbuminemia in older age are complex and multifactorial. Decreased hepatic synthesis may be secondary to hepatic impairment or the aging process itself [3,14]. Chronic inflammation secondary to underlying comorbidities such as metabolic syndrome and diabetes may also lead to lower serum albumin levels [15,16], as well as sarcopenia and malnutrition [17]. Moreover, hypoalbuminemia has been linked to the normal aging process as part of the natural decline of physiological reserve [13,17] and as a biochemical manifestation of frailty [12,13]. These overlapping mechanisms explain why hypoalbuminemia often reflects not just nutritional status, but also the cumulative effects of disease burden, inflammation, and frailty in the elderly. 

From a clinical perspective, these results have several implications. Serum albumin is a simple, inexpensive, and routinely available test that can be integrated into geriatric assessment tools to improve risk stratification. Identifying patients with significant hypoalbuminemia on admission could prompt early interventions, such as nutritional optimization, enhanced monitoring, and proactive management of comorbidities to potentially mitigate adverse outcomes. Albumin not only reflects nutritional status [18] but also correlates inversely with systemic inflammation and comorbidity burden [3], reinforcing its value as a multidimensional biomarker. With emerging evidence linking hypoalbuminemia to frailty [19], the prognostic significance of serum albumin levels should not be overlooked in geriatric populations.

Several limitations should be acknowledged. This was a retrospective, single-center study, which may limit generalizability. Despite adjustment for major confounders, unmeasured factors such as dietary protein intake, renal or hepatic protein loss, and specific comorbidities could have influenced albumin levels. The modest sample size may have reduced the statistical power to detect some associations, such as between albumin and readmission alone. Future research should confirm these findings in larger, multicenter cohorts and explore whether albumin-targeted interventions can translate into improved clinical outcomes.

## Conclusions

To conclude, our findings extend upon recent literature supporting the prognostic value of serum albumin in older patients. This study contributes new data in an intermediate care setting over a medium-term follow-up and suggests clinical strategies whereby albumin could be used as part of a multimodal assessment to better prognosticate older adults and potentially improve outcomes. The evidence underscores albumin’s role not only in nutritional assessment but also in predicting outcomes beyond frailty and multimorbidity. Prospective, multicenter studies are recommended to validate these findings and address the generalizability limitation. Further research should also examine whether interventions that raise albumin, via nutrition or other modalities, can improve outcomes in this population.
